# Kynurenine Monooxygenase Expression and Activity in Human Astrocytomas

**DOI:** 10.3390/cells10082028

**Published:** 2021-08-08

**Authors:** Gustavo Ignacio Vázquez Cervantes, Benjamín Pineda, Daniela Ramírez Ortega, Alelí Salazar, Dinora Fabiola González Esquivel, Daniel Rembao, Sergio Zavala Vega, Saúl Gómez-Manzo, Gonzalo Pérez de la Cruz, Verónica Pérez de la Cruz

**Affiliations:** 1 Neurochemistry and Behavior Laboratory, National Institute of Neurology and Neurosurgery “Manuel Velasco Suárez”, Mexico City 14269, Mexico; guigvace@gmail.com (G.I.V.C.); drmz_ortega@hotmail.com (D.R.O.); dinora.gonzalez@innn.edu.mx (D.F.G.E.); 2 Posgrado en Ciencias Biológicas, Unidad de Posgrado, Edificio A, 1° Piso, Circuito de Posgrados, Ciudad Universitaria, Mexico City 04510, Mexico; 3 Neuroimmunology Department, National Institute of Neurology and Neurosurgery “Manuel Velasco Suárez”, Mexico City 14269, Mexico; benpio76@hotmail.com (B.P.); ajsalazar27@gmail.com (A.S.); 4 Neuropathology Department, National Institute of Neurology and Neurosurgery “Manuel Velasco Suárez”, Mexico City 14269, Mexico; jdrbojorquez2002@gmail.com (D.R.); sergio.zavala.vega@gmail.com (S.Z.V.); 5 Laboratorio de Bioquímica Genética, Instituto Nacional de Pediatría, Secretaría de Salud, México City 04530, Mexico; saulmanzo@ciencias.unam.mx; 6 Department of Mathematics, Faculty of Sciences, Universidad Nacional Autónoma de México, UNAM, Mexico City 04510, Mexico; gonzalo.perez@ciencias.unam.mx

**Keywords:** tryptophan catabolism, kynurenine monooxygenase, glioblastoma

## Abstract

Glioblastoma multiforme (GBM) is the most common and aggressive primary brain tumor. The enzyme indoleamine-2,3-dioxygenase (IDO), which participates in the rate-limiting step of tryptophan catabolism through the kynurenine pathway (KP), is associated with poor prognosis in patients with GBM. The metabolites produced after tryptophan oxidation have immunomodulatory properties that can support the immunosuppressor environment. In this study, mRNA expression, protein expression, and activity of the enzyme kynurenine monooxygenase (KMO) were analyzed in GBM cell lines (A172, LN-18, U87, U373) and patient-derived astrocytoma samples. KMO mRNA expression was assessed by real-time RT-qPCR, KMO protein expression was evaluated by flow cytometry and immunofluorescence, and KMO activity was determined by quantifying 3-hydroxykynurenine by HPLC. Heterogenous patterns of both KMO expression and activity were observed among the GBM cell lines, with the A172 cell line showing the highest KMO expression and activity. Higher KMO mRNA expression was observed in glioma samples than in patients diagnosed with only a neurological disease; high KMO mRNA expression was also observed when using samples from patients with GBM in the TCGA program. The KMO protein expression was localized in GFAP^+^ cells in tumor tissue. These results suggest that KMO is a relevant target to be explored in glioma since it might play a role in supporting tumor metabolism and immune suppression.

## 1. Introduction

Glioblastoma multiforme (GBM) is an astrocyte-derived neoplasm and is the most frequent and aggressive of the primary brain tumors [1,2]. The standard care for GBM consists of surgical resection and radiotherapy plus concomitant chemotherapy with temozolomide, which provides a median overall survival of 18 months. Less than 10% of patients with GBM survive for more than 5 years [2,3,4], so GBM remains an incurable disease.

Over the past two decades, knowledge about cancer biology has been modeled around the “Hallmarks of Cancer”, a series of intrinsic properties of malignant cells that allow tumor formation [5,6]. Some of these properties allow malignant cells to uncontrollably proliferate and escape programmed cell death, while others allow malignant cells to modify their microenvironment in their favor, such as modulating the immune response, promoting angiogenesis, and promoting aberrant metabolic activity [5]. These properties could lead to the formation of a heterogeneous landscape not only by malignant cells that differ from each other, depending on their metabolic requirements, but also by the presence of endothelial cells and populations of the innate and adaptive immune system [6,7]. Tumor heterogeneity shaped by genetic and metabolic aberrations as well as by immunoediting mechanisms confers GBM high resistance to drugs, which leads to poor prognosis for the patient [8]. Therefore, the interconnections between two or more malignant cell hallmarks and the revelation of intercellular associations could offer novel insights into cancer biology, as well as promising targets for therapeutic strategies.

GBM presents metabolic and immune advantages [9,10,11,12,13] that favor the modeling of a heterogeneous tumor landscape, rapid growth rates, and evasion of anti-tumor immune responses [14,15,16,17]. One of these mechanisms of recent importance is the high expression of the enzyme indoleamine-2,3-dioxygenase (IDO). The presence of IDO in GBM tissue is associated with the infiltration of tumor-associated macrophages (TAMs) and regulatory T lymphocytes (Tregs) and with poor prognosis [18,19]. IDO participates in the initial and rate-limiting step of tryptophan catabolism through the kynurenine pathway (KP). N-folmylkynurenine is the cleavage product of tryptophan catabolism by IDO in extrahepatic tissue, which is rapidly converted to kynurenine (KYN) [20]. The catabolite KYN can be taken by kynurenine aminotransferase (KAT), kynureninase (KYNU), and kynurenine monooxygenase (KMO) to produce kynurenic acid (KYNA), anthranilic acid (ANA), and 3-hydroxykynurenine (3-HK), respectively. Then, 3-hydroxyanthranilic acid (3-HANA) is produced and leads to picolinic and quinolinic acid (QUIN) formation. Finally, quinolinic acid phosphoribosyl transferase (QPRT) produces the coenzyme NAD^+^ de novo [21]. The KP intermediary products are relevant for central nervous system homeostasis and the regulation of the immune response that may play a role in gliomagenesis [21,22].

Due to the clinical importance of IDO in GBM pathophysiology, studies have searched for pharmacological inhibitors or abrogation of IDO expression [23,24,25,26]. The use of the IDO inhibitor 1-methyl-tryptophan is related to tumor growth suppression and alone or in combination with temozolomide favorably affects the survival of glioma experimental models [24,25,27,28]. IDO inhibitors are also under investigation in clinical trials, but until now, there is no information about the results. Another enzyme of KP metabolism involved in many tumors, including malignant gliomas, is tryptophan dioxygenase-2 (TDO2) [29]. TDO2 is constitutively present in the liver and regulates the systemic levels of Trp, leading to KYN formation. A recent study showed that under normoxic conditions, TDO2 is expressed in GBM cells and suppresses T cell proliferation, while under hypoxic conditions, TDO2 expression is reversibly downregulated in GBM cells, restoring T cell proliferation, suggesting an immunomodulatory effect of TDO2 [30]. In accordance with this, KYN is shown to promote the differentiation of regulatory T cells through a mechanism involving AhR [31]. Additionally, 3-HANA and QUIN can inhibit T cell function and induce Th1 cell death [32,33]. However, the relevance of circulating tryptophan catabolites, such as KYN, and the expression of TDO2 and downstream KP enzymes in tumor pathophysiology are unclear.

The importance of KP components in immunosuppression has been demonstrated in other types of cancer; however, their role in malignant glioma cells is unclear. A recent study demonstrated that GBM cell lines can produce metabolites from the two branches of the KP [30]. In accordance with this, KMO leads to the long branch of the KP using KYN as a substrate to produce immunoregulatory tryptophan metabolites related to immune tolerance, such as 3-HK, 3-HANA, and QUIN [34,35,36,37]. KMO expression is present in macrophage and monocyte populations as well as in the microglia of the central nervous system (CNS) [38]. In these cells, KMO expression supports mitochondrial metabolism during pro-inflammatory polarization [39,40,41]. KMO expression remains restricted to microglia and some neuronal populations within the CNS under healthy conditions [42]. However, it has been postulated that the presence of the active KMO branch of the KP could represent an advantage for malignant cells in GBM [43,44]. Therefore, this work aimed to explore KMO expression and activity in different GBM cell lines as well as in tumoral tissue from patients with GBM.

## 2. Materials and Methods

### 2.1. Materials

Kynurenine (L-KYN), 3-hydroxykynurenine (3-HK), NADPH, glucose-6-phosphate (G6P), and glucose 6-phosphate dehydrogenase (G6PDH) were obtained from Sigma-Aldrich (St. Louis, MO, USA). Cyto-Fast Fix/Perm solution was obtained from BioLegend (San Diego, CA, USA). All other chemicals were of the highest commercially available purity. All solutions were prepared using deionized water obtained from a Milli-RQ (Millipore; Burlington, MA, USA) purifier system.

### 2.2. Glioblastoma Cell Lines

GBM cell lines U373, U87, LN18, and A172 (ATCC) were cultured in fresh DMEM (Dulbecco) supplemented with 10% fetal bovine serum and 1% streptomycin at 37 °C in an atmosphere of 5% CO_2_. The main genetic alterations for each cell line are shown in Table 1. For the experiments, the cells were collected and resuspended in Total RNA Isolation Reagent (TRIzol; Thermo Fisher Scientific, Waltham, MA, USA) for mRNA purification or in 1× PBS for KMO protein and enzyme activity determination.

### 2.3. Tumor Tissue Samples

Tumor samples were obtained from the National Institute of Neurology and Neurosurgery tumor bank (Mexico City, Mexico). The patients were diagnosed with different grades of astrocytoma without previous treatment and had enrolled for their first tumor resection between 2013 and 2014. Tumor samples kept in TRIzol were used for mRNA extraction and PCR, while samples kept in liquid N_2_ (freezing) were used to determine KMO activity. For immunofluorescence staining, paraffin-preserved tissue blocks from the same patients were obtained from the Department of Pathology of the National Institute of Neurology and Neurosurgery. A blinded expert pathologist determined the tumor grade. Data of the age, gender, overall survival (OS), and post-surgery treatment of each patient were obtained from the institute’s clinical files and are summarized in Results section.

### 2.4. KMO mRNA Expression

Total RNA was obtained from 1.5 × 10^7^ cells from TRIzol-conserved cell lines and patient samples according to the manufacturer’s specifications. Briefly, after phenol/chloroform separation, cold isopropanol was used for RNA precipitation. Samples were dried at room temperature and dissolved in pyrogen-free distilled water. cDNA was obtained using the First Strand cDNA Synthesis System for quantitative RT-PCR (RT-qPCR) (NP100042; OriGene, Rockville, MD, USA) according to the manufacturer’s specifications. The cDNA was kept at −20 °C before real-rime qRT-PCR. The SensiFAST SYBR Master Mix Kit (QP100016; OriGene) was used for RT-qPCR-based determination of KMO expression according to the manufacturer’s instructions. The KMO Human qPCR Primer Pair (HP 207154; OriGene) and GAPDH Human qPCR Primer Pair (HP205798; OriGene) were used, and their sequences are listed in Table 2. GAPDH was used as a housekeeping gene. A peripheral blood mononuclear-cell-derived DNA standard curve was used to quantify the mRNA copy number from the samples; data were expressed as the KMO mRNA copy number.

### 2.5. KMO Protein Expression

Cells were washed with 1× PBS, incubated with trypsin/EDTA solution, and then collected and permeabilized with 1× Cyto-Fast Fix/Perm solution for 30 min. Next, the cells were washed with 1× PBS, centrifuged at 2000 rpm for 5 min, and incubated with anti-KMO-middle region primary antibody (OAAB05255; Aviva, San Diego, CA, USA) for 30 min. After incubation, the cells were washed and centrifuged again, goat anti-rabbit IgG/Alexa 488 antibody (A11008; Invitrogen; Waltham, MA, USA) was added, and 10,000 total events were acquired. Finally, the KMO-positive mark, green fluorescence, was determined using a FACSCalibur flow cytometer and CellQuestPro software (Becton, Dickinson and Company, San Jose, CA, USA).

### 2.6. KMO Activity

KMO activity was measured in GBM cell lines (U87, U373, LN18, and A172) as well as in tumor tissue homogenates. Briefly, 2 × 10^6^ cells or 150 µL of homogenized tissues were diluted in 450 µL of KMO buffer (100 mM TRIS, 10 mM KCl, and 1 mM EDTA; Sigma-Aldrich, St. Louis, MO, USA). Then, 80 µL of the mixture was incubated with 100 µL of assay cocktail (1 mM NADPH, 3 mM G6P, 1 U/mL of G6PDH, and 100 µM KYN) in a final volume of 200 µL at 37 °C for 2 h. The reaction was stopped with 25 µL of 6% perchloric acid. Blanks were obtained by boiling the samples for 10 min. The samples were centrifuged at 14,000× *g* for 10 min. The samples were collected, and 3-HK was determined by HPLC using an electrochemical method [45]. Briefly, 100 µL of the supernatant was eluted at a constant flow rate of 0.5 mL/min with a mobile phase (0.59% phosphoric acid, 1.5% acetonitrile, 0.9% triethylamine, 0.27 mM EDTA, and 8.9 mM sodium heptane sulphonic acid) through an Adsorbosphere Catecholamine C18 reverse-phase column (3 μm, 4.6 mm × 100 mm; Thermo Fisher Scientific, Hampton, NH, USA). The oxidation voltage was 0.5 V at a range of 1.0 nA and a filter of 0.10 Hz (LC-4C detector; BAS). The retention time was ~11 min. The results are shown as pmoles of 3-HK/h/mg of protein.

### 2.7. Immunofluorescence Staining

For all cell lines, 3 × 10^4^ cells were seeded in 8-well slides in fresh culture medium and left overnight. Then, the cells were fixed in 100% methanol for 5 min. For the glioma tissue block, 5-µm-thick sections were cut, submerged in sodium citrate buffer (10 mM sodium citrate, 0.05% Tween 20, pH 6.0), and incubated in boiling water for 5 min for antigen retrieval. The sections were washed with 1× PBS and blocked for 30 min with 1% bovine serum albumin. After blocking, the sections were incubated with rabbit anti-KMO-middle region primary antibody (OAAB05255; Aviva, San Diego, CA, USA) for 30 min, washed, and incubated with mouse anti-rabbit IgG/Alexa Fluor 488 antibody (A11008; Invitrogen) for 30 min. The mouse anti-GFAP/PE primary antibody (sc-33673; BioLegend) was used to label astrocytic malignant cells. Images of the sections were obtained at 40× magnification using an OLYMPUS 1Χ81 microscope.

### 2.8. Protein Determination

Protein was determined according to Lowry’s method [46] using bovine serum albumin as a standard.

### 2.9. Statistics

Data of KMO activity were corrected by mg of protein. Results were expressed as the mean ± SEM. The Mann–Whitney test was performed to compare distributions of control and glioma groups using Prism software (GraphPad, San Diego, CA, USA). *p*-values < 0.05 were considered statistically significant. Survival time in each group was calculated using the Kaplan–Meier method and compared using the Mantel–Cox test.

### 2.10. Genomic Expression Analysis

The data were based on a combined cohort of the Cancer Genomic Atlas (TCGA) and the Genotype-Tissue Expression (GTEx) samples available on the Xena platform [47] and used in GEPIA2 [48]. The analysis included 207 brain cortex (non-diseased), 523 low-grade glioma, and 171 GBM samples. Expression values were plotted using Prism v 9.1.2 (GraphPad Software, La Jolla, CA, USA). Differences in gene expression levels between the groups were evaluated using the Kruskal–Wallis test, with Dunn’s test for pairwise comparisons. A *p*-value < 0.001 was considered statistically significant. Kaplan–Meier plots were used for survival analysis using the data from the GBM group; the patients were divided into two groups based on the median of KMO expression, and the log-rank test was performed to compare between the groups.

## 3. Results

### 3.1. Expression and Activity of Kynurenine Monooxygenase in Glioblastoma Cell Lines

**Figure 1:**
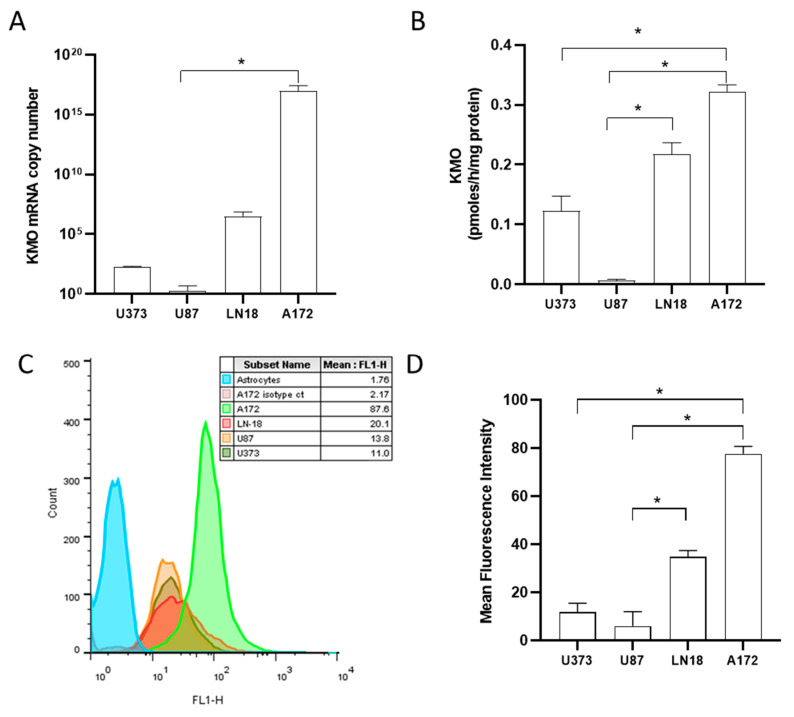
Kynurenine monooxygenase expression and activity in GBM cell lines. Quantification of KMO mRNA copies (**A**) and KMO activity (**B**) in GBM cell lines A172, LN-18, U87, and U373. Representative histogram (**C**) and KMO protein expression in GBM cell lines (**D**). Data in (**A**,**B**,**D**) are the mean ± SEM (*n =* 3–5); * *p <* 0.05 based on the Kruskal–Wallis test, with Dunn’s test for pairwise comparisons.

KMO leads to the KP branch that drives NAD^+^ formation, and KMO expression could represent a selective advantage for malignant cells. Thus, we quantified the KMO mRNA expression using real-time RT-qPCR in U87, U373, LN18, and A172 GBM cell lines. We found heterogeneous expression levels among the different cell lines, with A172 cells showing the highest KMO mRNA expression (Figure 1A). KMO protein expression among the different cell lines was different, with A172 cells again exhibiting the highest expression (Figure 1C,D). In addition to KMO mRNA and protein expression, KMO activity was analyzed in the cell lines. Figure 1B shows that all GBM cell lines exhibited KMO activity, with A172 cells showing the highest activity (0.322 ± 0.01 pmoles/h/mg protein), followed by LN18 and U373 cells. The U87 cell line showed less than 0.1 pmoles/h/mg protein expression, which, compared to KMO activity in peripheral blood mononuclear cells (2.9 ± 0.3 pmoles/h/mg protein), is low.

**Figure 2:**
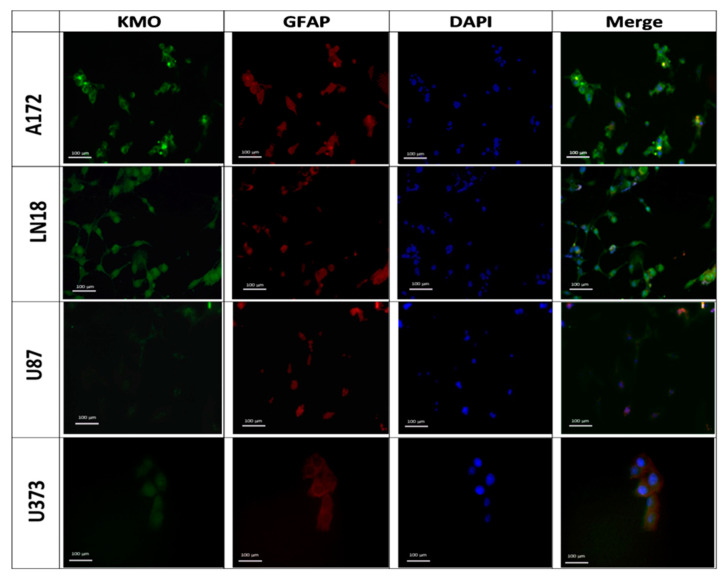
Kynurenine monooxygenase expression in GBM cell lines. Representative images of KMO protein expression in GBM cell lines A172, LN-18, U87, and U373. Images were acquired at 40× magnification. Scale bars represent 100 µm.

Furthermore, KMO protein expression was observed by immunofluorescence. Figure 2 shows that KMO protein was expressed in A172, LN18, and U373 cells, with A172 cells expressing more KMO protein than the other cell lines, which corresponds to the results in Figure 1D. Our results on GBM cell lines demonstrate that KMO is expressed and active in GBM malignant cells, but both mRNA and protein expression as well as KMO activity differ among the GBM cell lines.

### 3.2. Kynurenine Monooxygenase Expression in the GBM Tumor Mass

The next step was to quantify the KMO mRNA expression, KMO mRNA protein, and KMO activity in GBM tumor tissue. Tissue samples from patients (National Institute of Neurology and Neurosurgery, Mexico City, Mexico) diagnosed with neurological diseases (*n =* 6), non-astrocytic brain tumors (*n =* 1), diffuse astrocytoma (*n =* 2), and high-grade astrocytoma (*n =* 7) were analyzed. The clinical and demographic data of these patients are summarized in Table 3.

**Figure 3:**
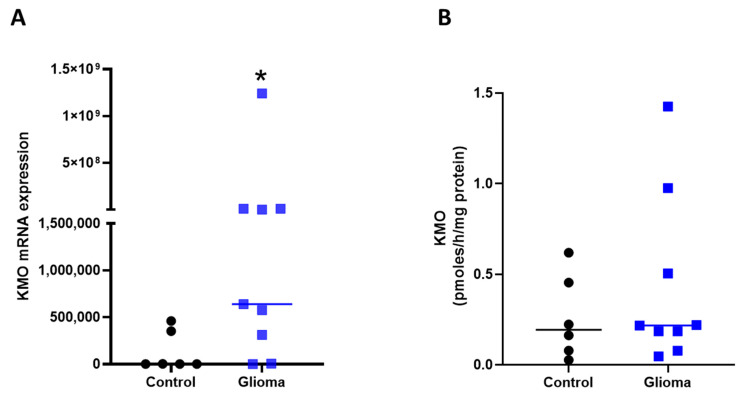
Kynurenine monooxygenase in GBM tissue. (**A**) KMO mRNA copy number in tissue samples: patients diagnosed with a neurological disease (control, *n =* 6) versus patients diagnosed with grade II to IV astrocytoma (glioma, *n =* 9). (**B**) Determination of KMO activity by quantification of 3-HK levels after 2 h incubation of the same samples used for KMO expression. Data are shown with the median (horizontal line); * *p <* 0.05 based on the Mann–Whitney test.

The KMO mRNA expression in tissue samples from patients with neurological diseases was significantly lower compared with patients with astrocytoma (0.13 × 10^6^ ± 0.086 × 10^6^ vs. 140.9 × 10^6^ ± 137.4 × 10^6^; *p* = 0.03; Figure 3A). However, no difference was observed in 3-HK levels (a product of KMO activity) between patients with neurological diseases and those with astrocytoma (Figure 3B).

**Figure 4:**
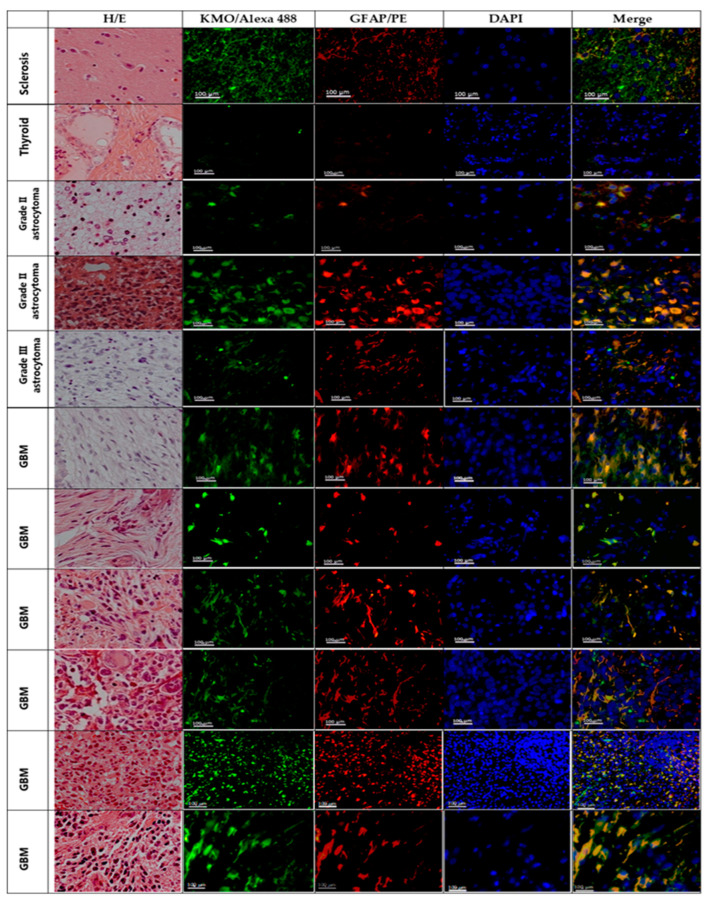
KMO in astrocytoma malignant cells. Representative images of hematoxylin/eosin (H/E) staining and immunofluorescence staining for KMO (KMO/Alexa Fluor 488; green), astrocytes or astrocytoma cells (GFAP/PE; red); and nuclei (DAPI; blue) in tissue samples from patients diagnosed with mesial sclerosis (line 1), thyroid carcinoma (line 2), and astrocytoma (lines 3–10). Images were acquired at 40× magnification. Scale bars represent 100 µm.

To ensure that the KMO mRNA expression and KMO activity in tumor tissue were due to the presence of KMO in malignant cells, KMO protein localization was determined by immunofluorescence staining (Figure 4, second column) in paraffin-embedded samples from the same patients analyzed by real-time RT-qPCR for KMO activity. Images of H/E-stained sections of tumor tissue were obtained to corroborate tissue architecture (Figure 4, first column). Glial fibrillary acidic protein (GFAP) immunofluorescence was used as an astrocytic lineage marker (Figure 4, third column). Tissue derived from a patient diagnosed with mesial sclerosis was used as a non-tumor tissue control, while that derived from a patient diagnosed with brain metastasis from thyroid carcinoma was used as a non-astrocytic tumor tissue. The mesial sclerosis sample showed KMO staining, where, in a few cases, GFAP^+^ cells were found (Figure 4, line 1). The thyroid carcinoma metastasis tissue did not show KMO and GFAP immunostaining (Figure 4, line 2). However, the astrocytoma tissue mainly localized KMO and GFAP in the same cells; furthermore, on some astrocytoma slides, KMO immunofluorescence was found unrelated to GFAP marking, suggesting a contribution to KMO expression in tumor-infiltrated cells (Figure 4, lines 3–10).

Additionally, we evaluated whether KMO mRNA expression could be an indicator of prognosis. The astrocytoma group was further divided into two groups, low KMO and high KMO, using the median KMO mRNA copy number. The survival time was considered an indicator of prognosis and compared between these two groups. However, no difference was observed (data not shown).

**Figure 5:**
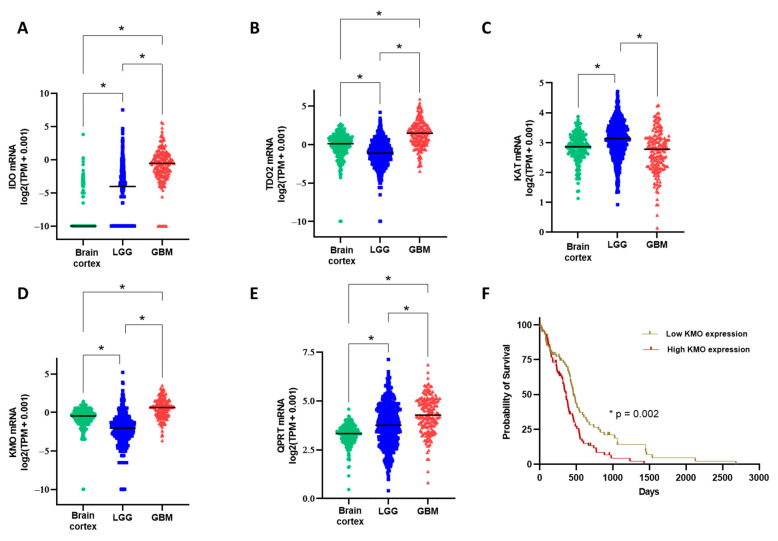
Comparison of gene expression levels (transcripts per million, TPM) on KP enzymes among three groups: brain cortex (non-diseased, GTEx project), low-grade glioma (TCGA program), and GBM (TCGA program). (**A**–**E**) mRNA expression of indoleamine dioxygenase (IDO), tryptophan dioxygenase (TDO), kynurenine aminotransferase II (KAT2), kynurenine monooxygenase (KMO), and quinolinic acid phosphoribosyl transferase (QPRT). * *p* < 0.001 based on the Kruskal–Wallis test, with Dunn’s test for pairwise comparisons. (**F**) Kaplan–Meier plot using the data from the GBM group; patients were divided into two groups based on the median KMO expression, and the *p*-value is based on the log-rank test.

In addition, the gene expression of KP enzymes (data from TCGA and GTEx programs) was analyzed. Figure 5 shows that IDO, TDO, KAT2, KMO, and QRPT were expressed in low-grade glioma and GBM, confirming our KMO experimental results. Furthermore, the KP enzyme expression in the GBM samples appeared to favor the long arm of the KP, which leads to QUIN and NAD^+^ production (Figure 5D,E). The GBM patients were further divided into two groups, low KMO and high KMO, according to the median KMO expression. The Kaplan–Meier plot and the log-rank test were used to assess the prognosis in these groups (Figure 5F). Results indicated that high KMO expression is associated with lower survival in patients with GBM.

## 4. Discussion

**Figure 6:**
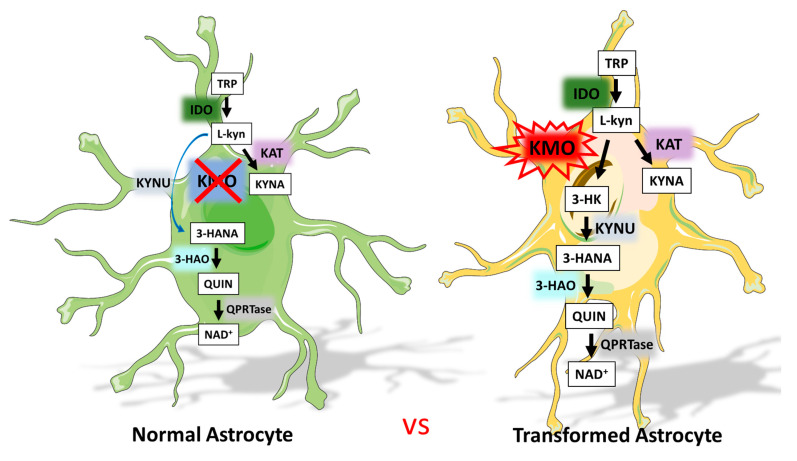
Tryptophan catabolism through the KP in normal astrocytes vs. transformed astrocytes.

Tryptophan catabolism through the KP in different kinds of cancers is receiving increasing attention because of its capacity to modulate the tumor immune environment. Specifically, IDO expression is related to GBM, but the rest of the KP in this pathology is still unclear. The relevance of the KP in GBM is related to the fact that kynurenine, the oxidation product of tryptophan, predominantly crosses the blood–brain barrier and can be degraded by both KP branches in the CNS, leading to immunomodulatory metabolites that can favor an immunosuppressive environment in GBM. This study showed for the first time that KMO, a critical enzyme of the KP, expressed on microglia in the CNS, is expressed and active in astrocytoma (Figure 6). The results showed heterogeneous KMO mRNA expression as well as differential KMO activity and protein expression in GBM cell lines, with A172 cells exhibiting the highest KMO expression and activity. The KMO expression and activity differences between GBM cells lines could be due to the distinct mutations in them (Table 1). It is noteworthy that the U87 cell line that showed the lowest KMO expression and activity did not show the p53 mutation according to the genomic profiles of several GBM cell lines [49]. Moreover, KMO was expressed and active in samples from patients diagnosed with astrocytoma of different grades. Interpatient heterogeneity was observed in this study, where a low/high KMO mRNA expression and activity pattern was found. However, the fact that KMO expression and activity are present in astrocytoma samples could be due to tumor-infiltrating cells, since KMO is highly expressed in immune cells, such as macrophages and monocytes and in CNS microglia [50]. Nevertheless, immunostaining for KMO showed that the protein is mainly localized to GFAP^+^ cells, indicating that tumor cells of the astrocytic lineage express KMO, but also in a heterogeneous way between different tumor samples. These data are in agreement with the data obtained by genetic analysis from TCGA and GTEx programs and from a recent study on patient-derived GBM cell lines that showed high heterogeneity in the expression of enzymes involved in the KP, including KMO [51].

These findings of KMO expression and activity in GBM cells represent a novel phenotypic characteristic that makes them different from normal astrocytes, which do not express KMO. In accordance with this, human fetal astrocytes were unable to produce 3-HK and KMO mRNA expression was not detected, even when they were stimulated with cytokines. However, KAT, 3-HAO, QPRT, and KYNU were expressed in human astrocyte cultures, indicating that KMO is the only KP enzyme that is not present in this cell type [52,53]. This change in KMO expression could be a consequence of the de-differentiation process described in glioma models that involves differentiated malignant cells gaining plasticity [54,55]. Furthermore, aberrant protein expression is related to tumorigenesis [56]. Our results are in agreement with a previous study that showed 3-HANA production in A172 cells under normoxic and hypoxic conditions, indicating that GBM malignant cells can produce KP downstream metabolites through KMO [30]. A recent study demonstrated that KMO is located in the cell membranes of canine mammary gland tumors as well as in human breast cancer samples. Furthermore, blocking the KMO surface with a KMO polyclonal antibody reduces migration and invasion of MDA-MB-231 cells [57]. Additionally, inhibition of KMO activity represses colorectal cancer cell migration, invasion, and tumor sphere formation [58].

There are three main hypotheses with regard to the relevance of active tryptophan catabolism through the KP in the tumor microenvironment [59]: first, overexpression of active IDO from malignant cells may deplete the tryptophan pool, thus inhibiting lymphocyte proliferation and abrogating the anti-tumor immune response [34]; second, active KP metabolism could favor the formation of tryptophan derivates that inhibit the proliferation of cytotoxic lymphocytes and induce their death [35,36]; and third, the KP can lead to the constant formation of NAD^+^, a coenzyme involved in metabolic and signaling pathways, contributing to the suitability of malignant cells [60]. In accordance with this, KMO is the pivotal enzyme in the KP that leads to NAD^+^ synthesis [61] and is related to poor prognosis in several cancers [58,62,63,64].

Our results support the heterogeneity of the KP within the GBM landscape; furthermore, in the cell lines tested, KMO is enzymatically active, and thus, tryptophan catabolism in GBM cells could lead to the formation of both KP metabolite immunoregulators and NAD^+^. Further analysis of the expression of enzymes such as QPRTase in GBM malignant cells will complement the findings of this study. The constant NAD^+^ synthesis in GBM cells supports the high metabolic and proliferative demand inherent in these tumors [65]. However, the differences in KMO expression and activity between the different GBM cell lines are also a reflection of the high heterogeneity of the GBM landscape [66]. Moreover, the presence of KMO activity in GBM cell lines could support the formation and accumulation of tryptophan catabolites, which have been described as toxic to immune populations, such as NK cells and T and B lymphocytes, since reports suggest that KP products 3-HK, 3-HANA, and QUIN decrease proliferation and increase apoptosis in these immune populations. Thus, the importance of KMO in GBM cells supports two of the main characteristics of the tumor microenvironment, namely metabolic activity and immune response modulation, making this enzyme a promising target for further study on GBM.

KMO expression in other types of cancer has also been explored. In this study, KMO mRNA expression did not affect patient survival, which could be due to the population size. However, KMO overexpression is related to malignancy and poor prognosis in patients with triple-negative breast cancer and colorectal cancer [58,62]. Furthermore, the oncogenic activity of KMO favors the potentiation of β-catenin signaling in an enzyme-activity-independent manner, indicating the role of KMO beyond 3-HK production [62]. KMO overexpression is also related to malignancy and poor prognosis in canine mammary gland tumors and melanomas. These studies show that KMO overexpression is parallel to signal transducer and activator of transcription 3 (STAT3) and pSTAT3 increase, which are related to the proliferation, survival, invasiveness, malignancy, and metastasis of tumor cells. In vitro pharmacological inhibition of KMO reduces tumor cell viability and STAT3 and pSTAT3 expression, confirming an oncogenic role of KMO outside of its catabolic activity [63,64]. In contrast, KMO expression increases in patients with advanced melanoma who show clinical benefit after PD-1 blockade therapy compared with PD-1-blockade-unresponsive patients [67]; however, the implications of KMO increase in these patients have not been discussed. Furthermore, the regulation of KMO expression could be crucial for the treatment of GBM, since in GBM cell cultures, KMO expression is upregulated after incubation with temozolomide but downregulated after incubation with the cyclin dependent kinase inhibitor, dinaciclib [51]. Nevertheless, the biological function of KMO in immunomodulation in glioblastoma and other cancers, as well as the association between KMO expression and the GBM genomic profile, should be clarified.

## 5. Conclusions

This study showed for the first time that KMO expression and activity are present in glioma cells. Assessing the importance of KMO in GBM and other cancers represents a novel field for understanding tumor cell biology with promising results. More information is needed to understand how KMO works in malignant cells as well as other types of cells that form a tumor mass. Therefore, future studies should focus on the dynamics of KP metabolism within CNS and immune cells.

## Figures and Tables

**Table 1:** Main genetic alterations of GBM cell lines.

Cell Line	U373	U87	LN18	A172
Main genetic alterations	p53 mutated PTEN mutated NF1 mutated EGFR amplification IDH^wt^	NF1 mutated IDH^wt^	p53 mutated PTEN mutated Chromosome p16 deletion IDH^wt^	p53 mutated PTEN deletion IDH (non-reported)

**Table 2:** Forward and reverse sequences of KMO and GAPDH primer pairs used for quantitative real-rime RT-qPCR.

Primer Pair	Sequence
KMO human	Forward: CGGATGCCATCCCTCTAATTGG Reverse: TGCATCTCCCAGCAGTACACAG
GAPDH human	Forward: GTCTCCTCTGACTTCAACAGCG Reverse: ACCACCCTGTTGCTGTAGCCAA

**Table 3:** Clinical and demographic features of patients.

	Neurological Disease	Non-Astrocytic Brain Tumor	Low-Grade Astrocytoma (Grade I/II)	High-Grade Astrocytoma (Grade III/IV)
*n*	6	1	2	7
Diagnosis	Mesial sclerosis (16%) Schizophrenia (16%) Facial paralysis (16%) Epilepsy (50%)	Metastasis from thyroid carcinoma (100%)	Diffuse astrocytoma (100%)	Anaplastic astrocytoma (14%) Glioblastoma multiforme (86%)
Sex (Men%/Women%)	50/50	0/100	0/100	43/57
Age (years)				
Mean ± SD	32 ± 19	40	47 ± 7	58 ± 12
Min.–Max.	20–64	40	42–53	23–75
Survival (days)				
Median	--	242	1298	137

## Data Availability

The data presented in this study are available upon request from the corresponding author.
